# Self‐Triggered Apoptosis Enzyme Prodrug Therapy (STAEPT): Enhancing Targeted Therapies via Recurrent Bystander Killing Effect by Exploiting Caspase‐Cleavable Linker

**DOI:** 10.1002/advs.201800368

**Published:** 2018-06-05

**Authors:** Seung Woo Chung, Jeong Uk Choi, Young Seok Cho, Ha Rin Kim, Tae Hyung Won, Peter Dimitrion, Ok‐Cheol Jeon, Seong Who Kim, In‐San Kim, Sang Yoon Kim, Youngro Byun

**Affiliations:** ^1^ Research Institute of Pharmaceutical Sciences College of Pharmacy Seoul National University Seoul 08826 South Korea; ^2^ Center for Nanomedicine Wilmer Eye Institute and Department of Ophthalmology Johns Hopkins University School of Medicine Baltimore MD 21231 USA; ^3^ Department of Molecular Medicine and Biopharmaceutical Sciences Graduate School of Convergent Science and Technology Seoul National University Seoul 08826 South Korea; ^4^ Boyce Thompson Institute and Department of Chemistry and Chemical Biology Cornell University Ithaca NY 14853 USA; ^5^ Pharosgen Co. Seoul 06034 South Korea; ^6^ Department of Biochemistry and Molecular Biology Asan Medical Center University of Ulsan College of Medicine Seoul 05505 South Korea; ^7^ Biomedical Research Institute Korea Institute of Science and Technology Seoul 02792 South Korea; ^8^ KU‐KIST school Korea University Seoul 02841 South Korea; ^9^ Department of Otolaryngology Asan Medical Center University of Ulsan College of Medicine Seoul 05505 South Korea

**Keywords:** bystander killing effects, cancer therapies, caspases, prodrugs, target therapies

## Abstract

Tumor heterogeneity is associated with the therapeutic failures of targeted therapies. To overcome such heterogeneity, a novel targeted therapy is proposed that could kill tumor populations with diverse phenotypes by delivering nonselective cytotoxins to target‐positive cells as well as to the surrounding tumor cells via a recurrent bystander killing effect. A representative prodrug is prepared that targets integrin αvβ3 and releases cytotoxins upon entering cells or by caspase‐3. This allows the prodrug to kill integrin αvβ3‐positive cells and upregulate caspase‐3, which in turn, activates the prodrug to release a cytotoxin that could subsequently diffuse into and kill the neighboring tumor cells. Apoptotic cells further upregulate and release caspase‐3, which activate more prodrugs leading to another round of adjacent cell death and caspase‐3 release. Thus, the bystander killing effect could occur repeatedly, leading to augmented and widespread anticancer activity. This strategy provides an avenue that could advance the current targeted therapy.

## 1 Introduction

For decades, targeted therapy has been regarded as a “magic bullet” that could selectively kill cancer cells and minimize damage to healthy cells. Thus, many different classes of targeted therapies are being developed and used clinically.^1^ Examples include antibodies, tyrosine kinase inhibitors (TKI), antibody–drug conjugates (ADC), and enzyme prodrug therapies (EPT).^1^, ^2^ However, despite the initial response, these targeted therapies failed to show enduring therapeutic outcomes in most patients.^3^ Tumor heterogeneity, the existence of distinct genetic, epigenetic, and phenotypic tumor cells between and within tumors, has been highlighted as one of the major reasons for the failure of targeted therapies.^4^ The genomic instability of cancer cells and the branching evolution that follows the Darwinian logic generate phenotypically diverse tumor subclones that could adapt to heterogeneous and dynamic tumor microenvironments, resulting in distinct subclones within a single tumor as well as between the primary tumor and distant recurrence.^5^ The highly selective nature of targeted therapy fundamentally limits its ability to combat heterogeneous cancers.

**Figure 1:**
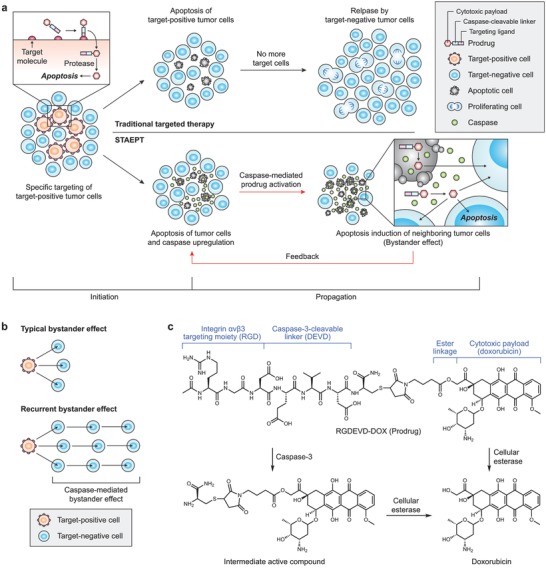
Self‐triggered apoptosis enzyme prodrug therapy (STAEPT). a) Schematic illustration depicting the mode of action of STAEPT and the b) difference between the typical bystander killing effect and the recurrent bystander killing effect of STAEPT. c) The chemical structure of RGDEVD‐DOX and its active compounds.

In this study, we propose a novel targeted therapy, self‐triggered apoptosis enzyme prodrug therapy (STAEPT), which is designed to deliver cytotoxic payloads not only to the target‐positive cells, but also to the surrounding cancer cells regardless of their phenotype by way of a recurring bystander killing effect, thereby affecting a broader population of cancer cells. The bystander killing effect, which has been demonstrated in ADC and EPT, is a phenomenon in which cell‐permeable cytotoxins released in targeted cells diffuse into neighboring cells leading to their death.^6^ There are two key events in the STAEPT paradigm: 1) the initiation step where target‐positive cells uptake the prodrug and metabolize it into an active compound, thus inducing their cell death; and 2) the propagation step where cell death leads to further activation of local prodrug thereby distributing cytotoxic payloads to adjacent cells in a recurrent manner (**Figure** **1**a). More specifically, the nonselective cytotoxic payload permeates cells by simple diffusion leading to the bystander killing effect and concurrently apoptosis‐associated proteases activate more local prodrugs, which enhances the bystander killing effect by allowing it to recur in a feedback loop (Figure 1b).

As a proof‐of‐concept, we prepared the model STAEPT prodrug, RGDEVD‐DOX, which contains a targeting domain, propagation domain, and nonselective cytotoxin. The peptide sequence RGD binds to integrin αvβ3, which is abundantly expressed on tumor endothelium as well as on several types of tumors,^7^ and acts as the targeting domain of our model STAEPT compound. Doxorubicin, a nonselective cytotoxin that initiates apoptosis,^8^ is conjugated to the αvβ3 targeting moiety by apoptotic caspase‐cleavable linker, which acts as the propagation domain. In particular, we selected apoptotic caspases as the protease to activate our prodrug, since cell death by cytotoxic drugs is frequently associated with the activation of apoptosis pathways in cancer cells.^9^ The core of the recurrent bystander killing effect is in the continual upregulation of a specific apoptosis‐associated protease, in this case caspase‐3, by cells killed in either the initial targeted manner or nonselective bystander killing. In this study, we demonstrate that STAEPT could achieve superior therapeutic benefit over existing targeted approaches while reducing toxic adverse effects by using our model prodrug.

## 2 Results

### 2.1 Design and Preparation of the Model Prodrug for STAEPT

RGDEVD‐DOX was prepared by conjugating doxorubicin to RGDEVD peptide via an ester linkage (Figure 1c). The peptide was designed to be the as short as possible, while still retaining bifunctionality. RGD and DEVD sequences with one Asp residue being shared by the two motifs produce the smallest possible bifunctional peptide. RGD is known to recognize integrin αvβ3 and has been popularly used to target tumor endothelial cells and several tumors.^10^ DEVD is a substrate sequence of a predominant apoptotic caspase, caspase‐3, where the amide bond following its last Asp residue is hydrolyzed when recognized by caspase‐3.^11^ In our prodrug, the DEVD motif plays the important role of the protease‐sensitive linker between RGD and the payload. This peptide is conjugated to doxorubicin via an ester linkage, which is cleaved by cellular esterase after entering the cells (Figure 1c).

### 2.2 Targeted Induction of Apoptosis by STAEPT Prodrug

**Figure 2:**
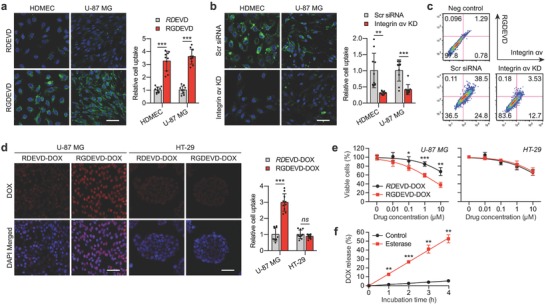
Enhanced cellular uptake and cytotoxic activity of RGDEVD‐DOX in integrin αvβ3 expressing cells. a) Representative confocal images (left) and quantitative analysis (right) of HDMEC and U‐87 MG cells exposed to fluorescent‐labeled RDEVD and RGDEVD peptides. b) Representative confocal images (left) and quantitative analysis (right) of scrambled control and integrin αv (ITGAV) siRNA‐transfected HDMECs and U‐87 MG cells exposed to fluorescent‐labeled RGDEVD peptide. Green and blue indicate the fluorescent‐labeled peptides and cell nuclei, respectively. Scale bar, 50 µm. c) Flow cytometry analysis of isotype control (top), scrambled control‐transfected (lower left), and ITGAV siRNA‐transfected (lower right) U87 MG cells incubated with fluorescent‐labeled RGDEVD and stained with an antibody against integrin αvβ3. d) Representative confocal images (left) and quantitative analysis (right) of U‐87 MG and HT‐29 cells treated with RDEVD‐DOX and RGDEVD‐DOX. Red and blue indicate the intrinsic red fluorescence of doxorubicin and cell nuclei, respectively. Scale bar, 50 µm. e) Concentration‐dependent cytotoxicity of RDEVD‐DOX and RGDEVD‐DOX on U‐87 MG (left) and HT‐29 (right) determined by MTT assay (*n* = 4). f) Doxorubicin release from RGDEVD‐DOX when incubated in PBS (pH 7.4) containing (or not containing) carboxylesterase (*n* = 3). Data are mean ± s.d. *P < 0.05, **P < 0.01, ***P < 0.001.

We investigated the integrin αvβ3 targeting capability of RGDEVD peptide in integrin αvβ3 overexpressing U‐87 MG and human dermal microvascular endothelial cells (HDMECs).^12^ Using confocal microscopy, we observed significantly higher cellular uptake of RGDEVD peptide in both cell lines than its analogous peptide (RDEVD) which lacks the proper targeting moiety (**Figure** **2**a). The transient knock down of integrin αvβ3 (Figure S1, Supporting Information) decreased the cellular uptake of RGDEVD peptide (Figure 2b), indicating that the cellular uptake was mediated by the interaction between the RGD motif and integrin αvβ3. This was further supported by flow cytometry, which showed decreased uptake of RGDEVD peptide in U‐87 MG after the knock‐down of integrin αvβ3 (Figure 2c).

The integrin αvβ3 targeting capability and the resulting cytotoxicity of our prodrug (RGDEVD‐DOX) and its RGD‐deficient analog (RDEVD‐DOX) were evaluated in U‐87 MG and HT‐29, which express integrin αvβ3 at high and low levels, respectively (Figure S2, Supporting Information).[[qv: 12b]] While we observed a significantly higher cellular accumulation of the prodrug than the analog in U‐87 MG, their degree of accumulation was similar in HT‐29, indicating that cellular uptake was facilitated by integrin αvβ3 (Figure 2d). Consequently, RGDEVD‐DOX exerted significantly higher cytotoxicity (IC_50_ = 2.51 × 10^−6^ m) than the RDEVD‐DOX analog (IC_50_ = 52.5 × 10^−6^ m) in U‐87 MG, whereas both compounds showed similar cytotoxicity in HT‐29 (IC_50_ = 55.5 and 42.54 × 10^−6^ m, respectively) (Figure 2e). The cytotoxic activity of the prodrug and the analog may be attributed to the release of the payload by cytoplasmic carboxylesterases after entering the cells. This was supported by our data that demonstrated the rapid release of doxorubicin from the prodrug in the presence of carboxylesterase (Figure 2f; Figure S3, Supporting Information).

### 2.3 STAEPT Prodrug Activation By Caspase‐3 Produces a Nonselective Cytotoxic Entity that Further Upregulates Caspase‐3 by Inducing Apoptosis in Target‐Positive and Target‐Negative Cells

**Figure 3:**
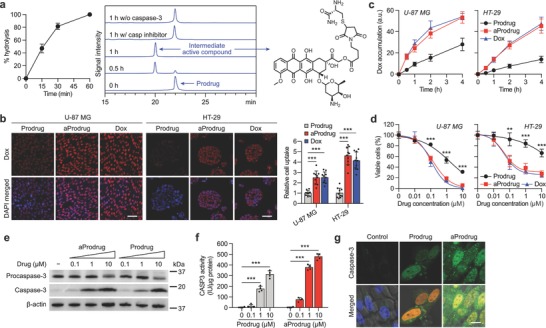
Caspase‐3‐mediated activation of the prodrug. a) HPLC chromatograms (center) and hydrolysis kinetic profile (left) of the prodrug incubated with caspase‐3 (*n* = 3). The HPLC chromatograms of the prodrug incubated without caspase‐3 or with caspase‐3 pretreated with caspase‐3 inhibitor are also presented. b) Representative images (left) and quantitative analysis (right) of U‐87 MG and HT‐29, each treated with the prodrug, activated prodrug (the prodrug incubated with caspase‐3; indicated as aProdrug), and doxorubicin. Scale bar, 50 µm. c) Cell accumulation kinetic profiles of the prodrug, activated prodrug, or doxorubicin in U‐87 MG (left) and HT‐29 (right). d) Concentration‐dependent cytotoxicity of the prodrug, activated prodrug, and doxorubicin on U‐87 MG (left) and HT‐29 (right) determined by MTT assay (*n* = 4). e) Western blot analysis of procaspase‐3 and caspase‐3. f) Quantitative cellular caspase‐3 activity analysis (*n* = 4) in U‐87 MG cells treated with increasing concentrations of the prodrug and activated prodrug. g) Intracellular caspase‐3 distribution of U‐87 MG cells after being exposed to the prodrug and activated prodrug. Blue, red, and green indicate the cell nuclei, intrinsic red fluorescence of doxorubicin, and caspase‐3, respectively. Data are mean ± s.d. **P < 0.01, ***P < 0.001.

The caspase‐3‐mediated activation of RGDEVD‐DOX was expected to release an intermediate molecule (Figure 1c), which will be referred to as “active compound” hereafter. The active compound may be eventually transformed into doxorubicin after entering the cell by the hydrolysis of the ester bond by intracellular carboxylesterases. The high‐performance liquid chromatography (HPLC) analysis of the prodrug incubated with caspase‐3 showed the appearance of a new peak with a mass similar to the predicted mass of the active compound (Figure S4, Supporting Information). More than 80% of the prodrug was converted into the active compound within 30 min in the presence of caspase‐3. However, such conversion was not observed in the absence of caspase‐3 or when the caspase‐3 was pretreated with a caspase inhibitor (**Figure** **3**a). The cellular accumulation of the activated prodrug was similar to the level of free doxorubicin in both U‐87 MG and HT‐29, suggesting that the cellular uptake was independent of integrin αvβ3 expression (Figure 3b,c). Consequently, the activation of the prodrug restored its cytotoxic activity to the level comparable to doxorubicin (IC_50_ = 0.113 and 0.243 × 10^−6^ m for U‐87 MG and HT‐29, respectively), decreasing the IC_50_ value from 1.62 to 0.146 × 10^−6^ m and from 49.0 to 0.375 × 10^−6^ m in U‐87 MG and HT‐29, respectively (Figure 3d).

Caspase‐3 expression and activity in response to the inactivated and activated prodrug were evaluated in U‐87 MG. The western blot results demonstrated concentration‐dependent upregulation of caspase‐3 by both the prodrug and the activated prodrug, where the activated prodrug was the more potent of the two (Figure 3e). Consistently, the quantitative caspase‐3 activity assay showed that the activated prodrug and the prodrug increased the cellular caspase‐3 activity 154‐fold and 99‐fold, respectively, at 10 × 10^−6^ m (Figure 3f). The upregulated caspase‐3 was shown to be distributed throughout the cells (Figure 3g). These data suggest that RGDEVD‐DOX is cleaved by caspase‐3 and the caspase‐3 activated prodrug leads to increased expression and activity of caspase‐3 via apoptosis. An earlier report demonstrated caspase‐3 is released from apoptotic cells,^13^ suggesting that prodrug approaching the tumor after the initial death of targeted cells could be further activated by secreted caspase‐3.

### 2.4 Selective Targeting and Caspase‐3‐Mediated Activation of the STAEPT Prodrug Individually Augmented the In Vivo Anticancer Activity

**Figure 4:**
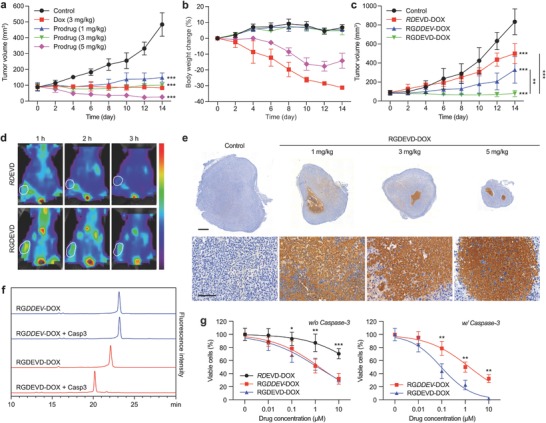
Anticancer activity of the prodrug in preclinical model. a) Tumor growth and b) body weight profiles of U‐87 MG xenografted mice that received normal saline as control, doxorubicin (3 mg kg^−1^), or the prodrug (1, 3, 5 mg kg^−1^ dox molar equivalent; *n* = 5). c) Tumor growth profiles of U‐87 MG xenografted mice that received normal saline as control, RDEVD‐DOX, RGDDEV‐DOX, or RGDEVD‐DOX (*n* = 6). d) Whole‐body fluorescent images of U‐87 MG xenografted mice that received NIR dye‐labeled RGDEVD or RDEVD peptides via tail vein. The circled region indicates the location of the tumor. e) Immunostaining of caspase‐3 in the tumor sections from (a). Scale bar, 1 mm (upper panels) and 100 µm (lower panels). f) HPLC chromatograms of RGDEVD‐DOX (red) and RGDDEV‐DOX (blue) incubated with or without recombinant human caspase‐3 for 60 min. g) Concentration‐dependent cytotoxicity of the prodrug and its analogs without (left) or with (right) pretreatment of recombinant human caspase‐3 in U‐87 MG cells determined by MTT assay (*n* = 4). Data are mean ± s.d. *P < 0.05, **P < 0.01, ***P < 0.001 versus control or as indicated.

Anticancer activity of RGDEVD‐DOX was investigated in the U‐87 MG xenograft model. Mice were dosed daily for seven days and clinical measures were monitored for 14 d (**Figure** **4**a; Figure S5a, Supporting Information). The treatment of the prodrug produced a dose‐dependent anticancer activity, with 1, 3, 5 mg kg^−1^ doses (dose are represented as doxorubicin molar equivalent hereafter) yielding 89, 97, and 114% tumor growth inhibition (TGI), respectively. The anticancer activity of the prodrug was comparable to that of doxorubicin, but with noticeably less toxicity, when administered at an equivalent dose (3 mg kg^−1^). While 3 mg kg^−1^ prodrug was well tolerated with no reduction in body weight, same dose of doxorubicin caused 30% reduction in body weight and 60% lethality. Despite higher efficacy, 5 mg kg^−1^ prodrug showed lower toxicity than 3 mg kg^−1^ doxorubicin, resulting in a 14% body weight reduction with no lethality (Figure 4b; Figure S6, Supporting Information). Although 5 mg kg^−1^ of the prodrug showed the highest anticancer activity, 3 mg kg^−1^ dose was selected for further animal studies, since it was the most well tolerated dose with potent anticancer activity.

The enhanced anticancer activities conferred by the RGD and DEVD moieties were investigated further in vivo by comparing the prodrug with its RGD‐deficient or DEVD‐deficient analog at a respective dose of 3 mg kg^−1^ (Figure 4c; Figure S5b, Supporting Information). All the analogs were expected to release the payload when they entered the cells by intracellular esterases similarly with the prodrug (Figure S3, Supporting Information). Our results demonstrated that while RGDEVD‐DOX produced 101% TGI, the RGD‐deficient analog RDEVD‐DOX produced 40% TGI (*P* < 0.0001), suggesting that the RGD augmented anticancer activity of the prodrug by its tumor targeting effect (Figure 4c). This was further supported by the ex vivo biodistribution imaging of U‐87 MG xenograft model that showed evident tumor accumulation of RGDEVD peptides but not RDEVD peptides (Figure 4d).

Immunohistochemical analysis of the tumors treated with the prodrug revealed substantial upregulation of caspase‐3 (Figure 4e; Figure S7, Supporting Information). To determine if the caspase‐3 cleavable linker, DEVD, improved the anticancer efficacy of our prodrug, we compared the prodrug with its DEVD‐deficient analog, RGDDEV‐DOX, which was prepared by rationally rearranging the conjugated peptide sequence rendering it immune to caspase‐3 mediated activation. The RGDDEV‐analog is identical in amino acid content, molecular weight, and retains the RGD‐mediated integrin αvβ3‐targeting capability (Figure S8, Supporting Information) but is unresponsive to caspase‐3 cleavage (Figure 4f). Due to the conserved RGD moiety, the resulting analog showed similarly enhanced cytotoxicity on U‐87 MG cells with RGDEVD‐DOX (IC_50_ = 1.47 and 1.24 × 10^−6^ m, respectively) when compared to the RGD‐deficient analog RDEVD‐DOX (IC_50_ = 65.4 × 10^−6^ m) (Figure 4g). Interestingly, despite similar in vitro cytotoxicity, RGDEVD‐DOX showed significantly higher in vivo anticancer activity than RGDDEV‐DOX, 101% versus 62% TGI (*P* = 0.0056) (Figure 4c). This suggests the caspase‐3‐mediated activation increases anticancer effect beyond RGD‐targeting of the prodrug. In fact, our in vitro study showed caspase‐3 augmented the cytotoxicity of RGDEVD‐DOX by an order magnitude (IC_50_ = 0.0962 × 10^−6^ m), it failed to elevate the cytotoxicity of RGDDEV‐DOX (IC_50_ = 1.51 × 10^−6^ m) (Figure 4g).

### 2.5 STAEPT Prodrug Shows Efficacy and Prolonged Survival in an In Vivo Model of Metastatic Lung Cancer

**Figure 5:**
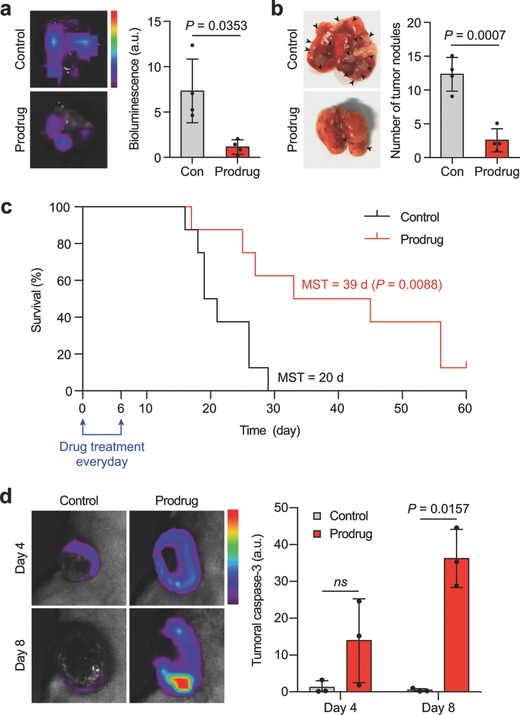
Anticancer activity of the prodrug in lung metastases. Representative images (left) and quantitative measurement (right) of a) bioluminescence and b) surface tumor nodules of the 4T1‐luc2 metastasized lung of mice treated with the prodrug. The arrowhead indicates tumor nodule. c) Kaplan–Meier survival curve of 4T1‐luc2 metastasis model that received normal saline as a control and the prodrug (*n* = 8). The blue square bracket indicates the drug treatment period. d) Representative bioluminescence images (left) and quantitative measurement (right) of caspase‐3 expression in the 4T1‐luc2 primary tumor at days 4 and 8 of mice treated with saline as a control and the prodrug (*n* = 3). Drug administration, 3 mg kg^−1^ dox molar equivalent once a day for seven days via the intravenous route. Data are mean ± s.d., ns: nonsignificant.

The anticancer activity of the prodrug was further evaluated in the highly invasive 4T1‐luc2 metastatic tumor model,^14^ which has low expression of integrin αvβ3 (Figure S2, Supporting Information). We observed immunostaining of integrin β3, which represents the expression of integrin αvβ3,^15^ mostly on the cells surrounding the blood vessels but rarely on the tumor cells in the metastatic lesion (Figure S9, Supporting Information), suggesting a heterogeneous population in terms of αvβ3 expression. Since tumor endothelial cells often overexpress integrin αvβ3,^16^ the integrin β3‐positive cells nearby the blood vessels were thought to be the tumor endothelial cells. The lung metastases were confirmed after two weeks of orthotropic inoculation of 4T1‐luc2 murine breast cancer cells (Figure S10a, Supporting Information). RGDEVD‐DOX was administered using the same dosing schedule described above. Decreased bioluminescence intensity (85% decrease) (**Figure** **5**a; Figure S10b, Supporting Information) and number of surface tumor nodules on the lung (82% decrease) (Figure 5b; Figure S10c, Supporting Information) demonstrate potent anticancer activity of RGDEVD‐DOX. Moreover, RGDEVD‐DOX significantly improved the survival time in comparison to untreated controls, almost doubling the median survival time (Figure 5c). This was achieved after only one week of daily treatment with the prodrug, in which the outcome could be improved by a more optimized dosing regimen consisting of repeated cycles of treatment.

We further investigated the caspase‐3 activity in the primary tumors of the 4T1‐luc2 model using an in vivo caspase‐3 detection probe throughout the course of treatment. We observed a substantial increase in caspase‐3 activity upon subsequent doses of RGDEVD‐DOX (Figure 5d; Figure S11, Supporting Information). Collectively, these data suggested that our prodrug initially induced apoptosis of tumor endothelial cells, followed by the upregulation of caspase‐3, which subsequently activated the prodrug in the tumor, suppressing the growth of metastases even though tumor cells had a low expression level of integrin αvβ3.

### 2.6 Enhanced Tumor Selectivity Significantly Reduced the Toxicity of the STAEPT Prodrug

**Figure 6:**
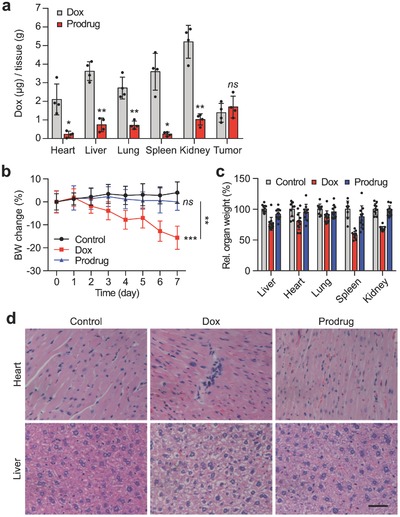
Tumor selectivity and toxicity of the prodrug in comparison to doxorubicin. a) Free doxorubicin concentration in normal organs and tumors of U‐87 MG xenografted BALB/c nude mice that received doxorubicin or the prodrug (*n* = 4). b) Body weight profiles, c) relative organ weights, and d) histopathological analysis of heart and liver tissues of normal ICR mice that received saline, doxorubicin, or the prodrug (*n* = 10). Representative H&E stained tissue sections are shown. Scale bar, 50 µm. Drug administration, 3 mg kg^−1^ dox molar equivalent once a day for seven days via the intravenous route. Data are mean ± s.d. *P < 0.05, **P < 0.01, ***P < 0.001 compared to control or as indicated. ns: non‐significant.

The potential toxicity of the prodrug, especially concerning the off‐target release of doxorubicin, was investigated after its administration of 3 mg kg^−1^ daily for seven days. The quantitative analysis of free doxorubicin in the tissue of U‐87 MG xenografted mice showed significantly lower doxorubicin accumulation in the normal organs of the prodrug treated group in comparison to the doxorubicin treated group, while doxorubicin accumulation in tumor was similar for the both groups (**Figure** **6**a).

In agreement with the above results, the prodrug demonstrated significantly reduced toxicities in healthy ICR mice when compared to doxorubicin. Doxorubicin is well‐known for causing severe adverse effects, with cardiotoxicity being the most prominent.^17^ Indeed, doxorubicin treatment resulted in severe reduction of body and organ weights (Figure 6b,c). The blood test results suggested the induction of cardiac and hepatic toxicities evidenced by increased creatine kinase‐muscle/brain (CK‐MB) and lactate dehydrogenase (LDH) levels, decreased glutamic‐pyruvic transaminase (GPT), blood urea nitrogen (BUN), total protein, and albumin levels (Table S1, Supporting Information). This was supported by histological examination that showed focal necrosis and cell infiltration in the heart and severe cellular edema in the liver (Figure 6d). The hematological analysis also suggested certain degree of myelosuppression evidenced by the decreased white blood cell and monocyte counts. However, our prodrug was well tolerated and showed no apparent toxicities except for marginal body weight loss and mild cellular edema in the liver.

## 3 Discussion

The importance of the selectivity of anticancer agents widely accepted since Paul Ehrlich postulated the concept of the “magic bullet”^1^ is challenged by notion of tumor heterogeneity and its role in the resistance against targeted therapies.^4^ Unlike targeted therapy, which is only effective on target‐positive cells, many cytotoxins are nonselective and more likely to kill diverse tumor populations with different phenotypes. Herein, we propose a new technology that combines the advantages of targeted and nonselective cancer therapies. In the STAEPT paradigm, nonselective cytotoxins are delivered by cancer homing entities to combat tumor heterogeneity while minimizing the deleterious side‐effects. The representative model prodrug RGDEVD‐DOX was prepared to target integrin αvβ3 and release the cell permeable and nonselective cytotoxic payload doxorubicin when encountered by caspase‐3. The RGD targets cancer by selectively binding integrin αvβ3, which is overexpressed in many cancers and tumor endothelial cells.^7^, ^10^ Upon binding, the cell takes up the prodrug and intracellular esterase releases the cytotoxic payload. This induces apoptosis of the target‐positive cell, which leads to an increase in the local caspase‐3 concentration. Caspase‐3 cleaves the prodrug releasing the cytotoxic entity, which enters adjacent cells by simple diffusion regardless of target expression. This again leads to apoptosis that further increases local caspase‐3, which subsequently triggers another cycle of prodrug activation and cellular apoptosis. The result is a recurrent bystander killing effect and activation of local prodrug and increased antitumor activity.

ADC and EPT have also been recognized to benefit from the bystander killing effect that occurs by the diffusion of cytotoxic payloads to target‐negative cells after being released in or on target‐positive cells, thus potentially killing the target‐negative cells besides the target‐positive cells.^6^ However, despite their superiority over traditional targeted therapies such as antibodies and TKIs in terms of their effectiveness on multiple tumor subclones, their bystander killing effect is completely dependent on the presence of target‐positive cells and nonrecurrent. Therefore, like the traditional targeted therapy, they would likely be ineffective when target‐positive cells are eliminated or absent. Since their principal target is target‐positive cells, those cells are likely to be eliminated earlier than the target‐negative cells, which would eventually result in resistance to the treatment. The STAEPT paradigm allows the treatment to continue to have efficacy even after the target‐positive cells are eliminated because it is not only initiated by the target‐positive cells, but also by dying cells that are generated repeatedly by the effects of nonselective cytotoxins, leading to more potent anticancer effect. In fact, the analog of our prodrug, RGDDEV‐DOX, that lacks the capability to be activated by caspase‐3, was significantly less effective than the prodrug. The analog was able to target integrin αvβ3 and release the payload in the cell by the action of intracellular esterase, thus it is expected to exert a bystander killing effect similar to ADC. This highlights the impact of the caspase‐mediated recurrent bystander killing effect contributing to the potent anticancer effect of our prodrug and potentially allowing it to overcome the current therapeutic challenges arose by tumor heterogeneity. This is particularly important regarding that tumor heterogeneity has been suggested as one of the major reasons for the clinical failure of many targeted therapies including targeted drug delivery systems that were proven successful in the preclinical models, in which the established tumors are mostly constituted of homogenous tumor cells unlike human tumors.^18^

Since the feedback activation of our prodrug was initiated from the tumor where target‐positive cells were located at the first place, we found the prodrug treatment resulted in remarkably decreased doxorubicin accumulation in the normal organs albeit similar accumulation in tumor when compared to free doxorubicin treatment, demonstrating a significantly improved therapeutic index. However, we acknowledge that there was some doxorubicin accumulation in normal organs and off‐target toxicity, especially when the prodrug was administered at a high dosage, which might be due to nonspecific uptake of the prodrug in the normal cells that led to the intracellular release of the payload. An alternative explanation for off‐target toxicity may be the hydrolysis of the prodrug by plasma carboxylesterases, which could also trigger the release of doxorubicin during circulation. These might also explain the anticancer activity shown by the prodrug analog with deficient RGD moiety (i.e., RDEVD‐DOX). However, while carboxylesterases are abundant in the plasma of rodents, this is not the case in humans.^19^ This suggests that the nonspecific hydrolysis of the prodrug during circulation would occur at a lower frequency in humans, thus lower toxicity when used clinically.

## 4 Conclusion

The STAEPT paradigm could be employed simply by inserting an apoptotic caspase‐cleavable or other cell death associated enzyme‐cleavable linker, followed by an additional linkage that could be cleaved upon entering the adjacent cells (e.g., ester or disulfide bond) between the targeting moiety and cytotoxic payload, improving the current targeted drug delivery strategies by producing the recurrent bystander killing effect. In fact, it has been suggested that the clinical failures of the attempts to selectively deliver drugs to the tumor that has been successful in preclinical models were due to the heterogeneous tumor in human patients while the tumors established in the preclinical models are largely homogeneous (ref). Moreover, Interestingly, DEVD has been reported to be hydrolyzed by cathepsin B at pH 5–6,^20^ suggesting that prodrug systems such as ADC that are internalized into the cells by endocytosis could easily take advantage of the STAEPT paradigm by simply replacing the linker with DEVD. In conclusion, we believe that STAEPT could be a promising strategy to improve upon current targeted therapies, especially the ones that exploit cytotoxins as their warhead.

## 5 Experimental Section

*Cell Lines*: HDMEC was purchased from PromoCell (Heidelberg, Germany). Human glioblastoma U‐87 MG and human colorectal adenocarcinoma HT‐29 were obtained from ATCC (Manassas, VA). Luciferase‐transfected murine breast carcinoma 4T1‐luc2 was obtained from PerkinElmer (Waltham, MA). HDMECs were cultured in Endothelial Cell Growth Medium MV2 (PromoCell). U‐87 MG, HT‐29, and 4T1‐luc2 cells were grown in high‐glucose Dulbecco's modified Eagle's medium (DMEM) (Gibco, Carslbad, CA) supplemented with 10% fetal bovine serum (FBS) (Gibco) and 100 U mL^−1^ penicillin–streptomycin (Gibco). The cells were maintained in a humidified 5% CO_2_ atmosphere at 37 °C. Human cancer cell lines were authenticated by STR DNA profiling and tested mycoplasma free.

*Synthesis*: Ac‐RGDEVDC‐NH_2_, Ac‐RDEVDC‐NH_2_, and Ac‐RGDDEVC‐NH_2_ peptides were purchased from Peptron (Daejeon, South Korea). Daunorubicin HCl was obtained from US Pharmacia (Rockville, MD). 1,4‐dioxane, anhydrous methanol, trimethyl orthoformate, propylene oxide, 48% hydrobromic acid, and 1 m potassium butoxide in tetrahydrofuran were purchased from Sigma‐Aldrich (St. Louis, MO). Bromine was obtained from Junsei Chemical (Tokyo, Japan). 4‐Maleimidobutyric acid was obtained from Santa Cruz Biotechnology (Dallas, TX). Cy5.5‐maleimide was obtained from Lumiprobe (Hallandale Beach, FL). All other solvents were from Burdick & Jackson (Seoul, South Korea).

14‐Doxorubicinyl maleimidobutyrate ester was synthesized according to Meyer‐Losic et al. for conjugation with the peptides.^21^ Daunorubicin HCl (100 mg, 177.3 µmol) was dissolved in a mixture of anhydrous methanol (3 mL) and anhydrous 1,4‐dioxane (2.5 mL). Trimethyl orthoformate (89.2 µL, 815.6 µmol, 4.6 eq.) was added, followed by the addition of bromine (15.7 µL, 306.8 µmol, 1.73 eq.) at 11 °C and reacted for 2 h under nitrogen. Propylene oxide (31.9 µL, 455.7 µmol, 2.57 eq.) was finally added at 4 °C and reacted for 75 min. Then the mixture of acetone (8.6 mL) and 0.25 m hydrobromic acid (3 mL) was reacted for 48 h at room temperature. When the reaction was completed, the solution was diluted with distilled water (5 mL) and extracted with chloroform (10 mL × 2). Saturated brine (5 mL) was added to the aqueous layer and the product was extracted into *n*‐butanol until the aqueous layer was colorless. The collected *n*‐butanol layer was concentrated at 35 °C in vacuo and precipitated in 10‐volume of *n*‐hexane to obtain 14‐halodaunorubicin as red solid. *m/z* (electrospray ionization mass spectrometry; ESI‐MS): 562.0 [M + H]^+^ for 14‐chlorodaunorubicin, 605.9 [M + H]^+^ for 14‐bromodaunorubicin.

Suspension of 4‐maleimidobutyric acid was prepared and 0.1 m sodium bicarbonate was slowly added while stirring. The resulting solution was stirred for 20 min and concentrated at 30 °C in vacuo. The concentrated solution was lyophilized to obtain sodium 4‐maleimidobutyrate. The sodium 4‐maleimidobutyrate (263 mg, 1.28 mmol) and 14‐halodaunorubicin (138 mg, 237.2 µmol) were dissolved in acetone and refluxed under nitrogen for 4 h. The solution was cooled and filtered. The remaining solid was washed with acetone and the filtrate was evaporated under vacuum. The red residue was dissolved in water and subjected to semi‐preparative reverse‐phase HPLC (Shimadzu, Kyoto, Japan) using an octadecylsilyl (ODS‐A) 5 µm semi‐preparative column (150 × 30 mm; YMC, Dinslaken, Germany) for further purification to obtain highly purified 14‐doxorubicinyl maleimidobutyrate ester. A gradient system (water and acetonitrile with 0.05% trifluoroacetic acid (TFA) as an additive) was used with a flow rate of 8 mL min^−1^. Each step of the reaction was monitored using a normal phase thin‐layer chromatography (TLC) (CH_2_Cl_2_:MeOH, 8:2). *m/z* (ESI‐MS): 709.0 [M + H]^+^. ^1^H NMR (500 MHz, MeOH‐*d*_4_, δ ppm): 7.98 (d, *J* = 7.8 Hz, 1H, H1), 7.85 (dd, *J* = 7.8, 7.8 Hz, 1H, H2), 7.59 (d, *J* = 7.8 Hz, 1H, H3), 6.80 (s, 1H, H6″, H7″), 5.46 (d, *J* = 2.8 Hz, 1H, H1′), 5.31 (d, *J* = 18.0 Hz, 1H, H14), 5.12 (br s, *J* = Hz, 1H, H7), 5.08 (d, *J* = 18.0 Hz, 1H, H14), 4.32 (q, *J* = 6.5 Hz, 1H, H5′), 4.03 (s, 1H, OCH3), 3.66 (br s, 1H, H4′), 3.58 (t, *J* = 6.5 Hz, 1H, H4″), 3.57 (m, 1H, H3′), 3.11 (d, *J* = 18.0 Hz, 1H, H10), 2.96 (d, *J* = 18.0 Hz, 1H, H10), 2.44–2.50 (m, 3H, H8, H2″), 2.03–2.10 (m, 2H, H8, H2′), 1.93 (tt, *J* = 6.5, 6.5 Hz, 2H, H3″), 1.87 (dd, *J* = 3.5, 13.0 Hz, 1H, H2′), 1.31 (s, 3H, H6′).

One of the selected peptides was reacted overnight with the prepared 14‐doxorubicinyl maleimidobutyrate ester (2 eq.) in anhydrous dimethylformamide (DMF) at ambient temperature. The solvent was removed in high vacuum and the red residue was redissolved in water. The crude product was purified using a semi‐preparative reverse‐phase HPLC as described above. The collected fraction was lyophilized to obtain final product as red powder. The purity was confirmed by analytical HPLC with UV detection at 214 nm and was determined to be ≥95%. High‐resolution mass spectrometry (HRMS) (electrospray ionization‐quadrople time of flight (ESI‐QTOF)) of RGDEVD‐DOX: calcd. for C_66_H_87_N_13_O_28_S, 1541.5504; found 1541.5514 (∆ = 0.62 ppm). RDEVD‐DOX: calcd. for C_64_H_84_N_12_O_27_S, 1484.5290; found 1484.5301 (∆ = 0.79 ppm). RGDDEV‐DOX: calcd. for C_66_H_87_N_13_O_28_S, 1541.5504; found 1541.5526 (∆ = 1.4 ppm). ^1^H NMR (500 MHz, MeOH‐*d*_4_, δ ppm) of RGDEVD‐DOX: 8.03 (d, *J* = 8.0 Hz, 1H, H1), 7.87 (dd, *J* = 8.0, 8.0 Hz, 1H, H2), 7.61 (d, *J* = 8.0 Hz, 1H, H3), 5.48 (br s, 1H, H1′), 5.32 (m, 1H, H14), 5.17 (br s, 1H, H7), 5.13 (m, 1H, H14), 4.66–4.72 (m, 2H, H13″, H27″), 4.61 (dd, *J* = 5.5, 8.5 Hz, 1H, H10″), 4.27–4.36 (m, 3H, H5′, H22″, H33″), 4.05 (s, 3H, OCH3), 4.00–4.04 (m, 2H, H6″, H17″), 3.87 (br s, 2H, H31″), 3.67 (s, 1H, H4′), 3.53–3.63 (m, 3H, H3′, H4″), 3.49 (m, 1H, H9″), 3.22 (m, 1H, H7″), 3.21 (m, 2H, H36″), 3.20 (d, *J* = 18.2 Hz, 1H, H10), 3.08 (d, *J* = 18.2 Hz, 1H, H10), 3.02 (dd, *J* = 8.5, 14.0 Hz, 1H, H9″), 2.76–2.97 (m, 4H, H14″, H28″), 2.41–2.52 (m, 6H, H8, H2″, H7″, H24″), 2.13 (m, 2H, H8, H18″), 2.08 (m, 2H, H2′, H23″), 2.01 (s, 3H, H38″), 1.83–1.97 (m, 4H, H2′, H3″, H34″), 1.59–1.74 (m, 3H, H34″, H35″), 1.32 (s, 3H, H6′), 0.95 (s, 6H, H19″, H20″).

The fluorescent labeled‐peptides were also prepared using a similar procedure. One of the selected peptides (1 eq.) was reacted overnight with Cy5.5‐maleimide in anhydrous DMF in the dark at 4 °C. The product was precipitated in diethyl ether, collected, washed, and dried in vacuo to obtain a dark blue crude product. The solid was dissolved in small volume of DMF and subjected to semi‐preparative reverse‐phase HPLC for further purification as described above. The purity of the obtained RGDEVD‐Cy5.5 and RDEVD‐Cy5.5 was confirmed by analytical HPLC with UV detection at 214 nm and was determined to be ≥95%. No peak corresponding to Cy5.5‐maleimide was detected, indicating the complete removal of unreacted dye.

*siRNA Transfection*: Transient gene knockdown of HDMEC and U‐87 MG was carried out using siRNA duplex against ITGAV (ITGAV Trilencer‐27 human siRNA; OriGene, Rockville, MD; cat. no. SR302468) or a scrambled negative control siRNA (OriGene; Cat. No. SR30004) following the manufacturer's instruction. Briefly, when the cells were almost confluent in 30 mm dish, 5 µL of siRNA duplex solution (5 × 10^−6^ m) was transferred to 140 µL of Opti‐MEM (Gibco). Transfection agent solution was prepared by the addition of siTran1.0 (20 µL; OriGene) to Opti‐MEM (120 µL). Both solutions were combined, incubated for 15 min at room temperature, and added to cells in a complete medium (final volume 2.5 mL) to gain a final concentration of 10 × 10^−9^ m siRNA. The ratio between siRNA and siTran1.0 was maintained for other concentrations of siRNA transfection. After 72 h incubation, the RNAi efficiency was confirmed by western blot analysis using anti‐integrin αv antibody (1:1000; Cell Signaling Technology, Danvers, MA). Further experiments were performed using the cells transfected with 10 × 10^−9^ m of ITGAV or scrambled siRNA duplex.

*Cellular Uptake Imaging*: HDMECs and U‐87 MG cells were seeded on glass bottom dishes (3 × 10^5^) and maintained until the cells reached 80% confluence. They were then treated with RGDEVD‐Cy5.5 or RDEVD‐Cy5.5 (1 × 10^−6^ m) and incubated for 5 min. For another set of experiments, ITGAV siRNA‐transfected and mock‐transfected HDMEC and U‐87 MG were prepared as described above, treated with RGDEVD‐Cy5.5 (1 × 10^−6^ m), and incubated for 5 min. Cellular uptake of the prodrug and its analogs and the prodrug pretreated with recombinant human caspase‐3 (500 ng mL^−1^; R&D Systems, Minneapolis, MN), where successful hydrolysis was confirmed by HPLC analysis, was also evaluated on U‐87 MG and/or HT‐29 cells. When the cells reached 80% confluence, each agent was treated (10 × 10^−6^ m) and incubated for an hour. The cells were washed with cold phosphate buffered saline (PBS), fixed with 4% paraformaldehyde (PFA), and mounted using SlowFade gold antifade reagent with 4′,6‐diamidino‐2‐phenylindole (DAPI) (Molecular Probes, Eugene, OR). The cells were observed under a confocal laser‐scanning microscope (LSM 710; Carl Zeiss, Oberkochen, Germany).

*Flow Cytometry*: U‐87 MG cells were transfected with ITGAV and scrambled control siRNA to produce integrin αv knocked down and negative control cells, respectively, as described above. Then, RGDEVD‐Cy5.5 (1 × 10^−6^ m) was treated for 5 min and washed with cold PBS. The cells were then detached with Trypsin/ethylenediaminetetraacetic acid (EDTA) (Gibco) and collected by centrifuge at 400 × g for 5 min. The cells were fixed in 2% PFA for 10 min at room temperature, washed, and suspended in PBS (100 µL) containing 0.5% bovine serum albumin (BSA) (Sigma‐Aldrich). The cells were incubated with Alexa Fluor 488‐conjugated integrin αvβ3 antibody (1:100; R&D Systems, Minneapolis, MN; Cat. No. FAB3050G) for an hour at 4 °C, washed, and suspended in PBS containing 0.5% BSA. The flow cytometry data were acquired using BD FACSCalibur (BD Biosciences, San Jose, CA) and processed using FlowJo software (FlowJo, LLC, Ashland, OR).

*Cytotoxicity Assay*: U‐87 MG and HT‐29 were seeded in a 96‐well plate (5 × 10^4^ per well) and incubated for 24 h. The cells were treated with the prodrug and its analogs and the prodrug pretreated with recombinant human caspase‐3 (500 ng mL^−1^) at various concentrations (*n* = 4) for an hour. The cell medium was replaced with fresh medium and further incubated for 72 h. 3‐(4,5‐Dimethylthiazol‐2‐yl)‐2,5‐diphenyltetrazolium bromide (MTT) assay was performed by the addition of MTT reagent (10 µL; Trevigen, Gaithersburg, MD) to each well followed by 2 h incubation at 37 °C. Then, a detergent agent (100 µL; Trevigen) was added to each well and further incubated for 4 h at room temperature. The absorbance was measured using a microplate reader (Synergy HT, BioTek Instruments, Winooski, VT) at 570 nm. A four‐parameter logistic curve fitting and the calculation of absolute IC_50_ were performed using GraphPad Prism 7.0a (GraphPad Software, San Diego, CA).

*HPLC Analysis of Carboxylesterase‐Mediated and Caspase‐Mediated Hydrolysis*: The carboxylesterase‐mediated doxorubicin release from the prodrug and the analogs was determined by incubating each compound (100 × 10^−6^ m) in PBS (pH 7.4; Gibco) containing carboxylesterase (30 U mL^−1^; Sigma‐Aldrich) at 37 °C. The caspase‐3‐mediated hydrolysis was determined by incubating each compound (100 × 10^−6^ m) with recombinant human caspase‐3 (500 ng mL^−1^) in a caspase assay buffer (Enzo Life Sciences, Farmingdale, NY) at room temperature. The reaction was quenched by the addition of an equal volume of dimethylsulfoxide (DMSO) at 30 and 60 min of incubation. The prodrug incubated for 1 h without caspase‐3 and with caspase‐3 (500 ng mL^−1^) pretreated with caspase‐3 inhibitor (Ac‐DEVD‐CHO, 10 × 10^−6^ m; Enzo Life Sciences) was also prepared. The samples were subjected to an analytical HPLC (Agilent 1300 series, Agilent Technologies, Santa Clara, CA) equipped with an ODS‐A 5 µm analytical column (150 mm × 3 mm; YMC). A gradient system (Water and ACN with 0.1% TFA as an additive, ACN 20–30%/5–25 min) was applied at a flow rate of 1 mL min^−1^. The peaks were monitored under a fluorescence detector at 470/580 nm.

*Western Blots*: To determine the caspase‐3 upregulation after treatment of RGDEVD‐DOX and activated RGDEVD‐DOX, U‐87 MG (80% confluency) was treated with different concentrations of each agent and incubated for 48 h. The cells were collected by centrifugation, washed with cold PBS, and lysed with radioimmunoprecipitation assay (RIPA) buffer (Pierce, Rockford, IL) containing protease inhibitor cocktail (Pierce). Untreated U‐87 MG, HT‐29, 4T1‐luc2, and ITGAV siRNA transfected HDMECs and U‐87 MG cells were also lysed to determine the expression of integrin αvβ3. The preparation of the samples and western blot were performed according to the standard protocol. The membranes were blotted with antibodies against procaspase‐3 (1:1000; cat. no. 9665), cleaved caspase‐3 (1:1000; cat. no. 9664), integrin αv (1:1000; cat. no. 4711), integrin β3 (1:1000; cat. no. 13166), and β‐actin (1:2000; cat. no. 3700), which were purchased from Cell Signaling Technology (Danvers, MA). The blotted membranes were developed under the ImageQuant LAS 4000 imaging system (GE Healthcare Life Sciences, Uppsala, Sweden).

*Cellular Caspase‐3 Activity Determination*: The cellular caspase‐3 activity was determined using the caspase‐3 cellular activity assay kit (EMD Millipore, Darmstadt, Germany) according to the manufacturer's instruction. Briefly, U‐87 MG (80% confluency) was treated with RGDEVD‐DOX or activated RGDEVD‐DOX at different concentrations and incubated for 48 h. The cells were collected by centrifugation, washed with cold PBS, and lysed with the cell lysis buffer included in the assay kit. The lysates were centrifuged at 14 000 rpm for 15 min using a refrigerated centrifuge, and the supernatant was collected. Every sample was adjusted to have equal protein concentration, and the caspase‐3 activity was determined.

*Cellular Caspase‐3 Staining*: U87 MG cells were seeded in 8‐well cover glass chamber (Nalge Nunc, Rochester NY) at seeding density of 1 × 10^5^ cells per well and grown until 80% confluence. RGDEVD‐DOX or activated RGDEVD‐DOX was treated to the cells at a final concentration of 1 × 10^−6^ m and incubated for 48 h. The activated caspase‐3 within the cells was stained using Image‐iT LIVE Green Caspase‐3 Detection Kit (Molecular Probes) according to the manufacturer's instruction. The kit is based on a fluorescent inhibitor of caspases (FLICA) methodology, essentially an affinity label. Briefly, FAM‐DEVD‐FMK reagent was added to the cells and incubated for 60 min. Then, the cells were further stained with Hoechst 33342 (included in the kit). The cells were washed twice with the provided wash buffer and fixed with 4% paraformaldehyde. The fixed cells were then observed under a confocal laser‐scanning microscope (LSM 710).

*Determination of In Vivo Anticancer Activity*: All experimental procedures were approved by the Institutional Animal Care and Use Committee of the Seoul National University. First, the anticancer activity of the prodrug in the established tumor of U‐87 MG was determined. For this, U‐87 MG cells (1 × 10^7^) were subcutaneously inoculated into the dorsal flank of 6‐week‐old male BALB/c‐nude mice (Orient Bio, Seongnam, South Korea). When the tumor volume reached 50–100 mm^3^, the mice were randomly grouped (*n* = 5 or 6) and given one of the following via tail vein once a day for seven days: normal saline, doxorubicin (3 mg kg^−1^), RGDEVD‐DOX (1, 3, 5 mg kg^−1^), RDEVD‐DOX (3 mg kg^−1^), and RGDDEV‐DOX (3 mg kg^−1^). Tumor size was measured using a digital Vernier caliper and the volume was calculated by the modified ellipsoid volume formula: *V* = (*a* × *b* ^2^)/2, where *V* is the tumor volume, *a* is the length (large diameter) of the tumor, and *b* is the width (small diameter) of the tumor. Percent tumor growth inhibition (%TGI) was determined by the following formula: %TGI = {1 − (*T*_t_/*T*_0_)/(*C*_t_/*C*_0_)}/{1 − (*C*_0_/*C*_t_)} × 100, where *T*_t_ is the median tumor volume of the treated group at the end of the study, *T*_0_ is the median tumor volume of treated group at the start of the study, *C*_t_ is the median tumor volume of the control group at the end of the study, and *C*_0_ is the median tumor volume of control group at the start of the study.

The anticancer effect of RGDEVD‐DOX in lung metastases was also evaluated. For this, 4T1‐luc2 cells (1 × 10^6^) were inoculated into the mammary fat pad of 6‐week‐old female BALB/c‐nude mice. When lung metastasis was confirmed by bioluminescence imaging using the Optix MX3 imaging system (Advanced Research Technologies, Montreal, Canada) after two weeks of inoculation, the mice were randomly grouped (*n* = 4) and given normal saline or RGDEVD‐DOX (3 mg kg^−1^) once a day for seven days via tail vein. A day after the last treatment, the mice were injected with D‐luciferin (2 mg) intraperitoneally 5 min prior to the removal of lung. The harvested lung was observed under Optix MX3 imaging system, and the bioluminescence intensity was quantified using the Image J software (NIH, Bethesda, MD). The tumor nodule on the surface of the lung was also counted. The survival analysis after treatment of RGDEVD‐DOX in the 4T1‐luc2 lung metastases model was performed in an independent study. The 4T1‐luc2 was inoculated to the mice as described above and grown for two weeks, followed by the surgical removal of the primary tumor. The mice were randomly grouped (*n* = 8) and given normal saline or RGDEVD‐DOX (3 mg kg^−1^) for once a day for seven days via tail vein starting from one day after the primary tumor removal. The doses of the doxorubicin conjugates were presented as molar equivalent dose of doxorubicin.

*Immunohistochemistry*: The formalin‐fixed paraffin‐embedded tissue specimens sectioned at 4 µm thickness were immunostained with primary antibodies against cleaved caspase‐3 (1:300; Cell Signaling Technology, cat. no. 9664) or integrin β3 (1:250; Cell Signaling Technology, cat. no. 13166). The horseradish peroxidase (HRP) visualization system (Dako, Carpinteria, CA) and 3,3′‐diaminobenzidine (DAB) were used in tandem to detect the primary antibody. The sections were then counterstained with hematoxylin. The tissue sections were imaged using Vectra 3 automated quantitative pathology imaging system (PerkinElmer).

*Whole‐Body Fluorescent Imaging*: U‐87 MG cells (1 × 10^6^) were inoculated on the left thigh of 6‐week‐old male BALB/c‐nude mice and grown until the tumor volume reached 500 mm^3^. Then, the mice were administered with RGDEVD‐Cy5.5 or RDEVD‐Cy5.5 (2 µmol kg^−1^) via tail vein (*n* = 1) and observed under Optix MX3 imaging system to visualize the whole‐body fluorescent images at predetermined time points.

*Bioluminescent Imaging of Caspase‐3 Upregulation In Vivo*: 4T1‐luc2 cells (1 × 10^6^) were inoculated into the mammary fat pad of 6‐week‐old female BALB/c‐nude mice. When the tumors reached 300 mm^3^, normal saline or RGDEVD‐DOX (3 mg kg^−1^) was administered daily via tail vein (*n* = 3). At day four and day eight of the first treatment, Z‐DEVD‐Aminoluciferin (VivoGlo Caspase‐3/7 substrate, Promega, Madison, WI) was administered intraperitoneally (5 mg), and bioluminescence indicating the expression of caspase‐3 was visualized after 10 min of injection under the Optix MX3 imaging system. The bioluminescence intensity was quantified using the Image J software.

*Doxorubicin Quantification in Tissues*: The determination of doxorubicin concentration in tissue samples was performed according to previous literature.^22^ U‐87 MG (1 × 10^7^) were subcutaneously xenografted into the dorsal flank of 6‐week‐old male BALB/c‐nude mice (Orient Bio). When tumor volume reached 300 mm^3^, the mice received doxorubicin or RGDEVD‐DOX once a day for seven days via intravenous route at 3 mg kg^−1^ doxorubicin molar equivalent dose (*n* = 4). A day after the last treatment, the organs and tumors were harvested. 200 µL of 20 × 10^−3^ m phosphate buffer (pH 3.8) was added per 50 mg of tissue sample and homogenized using a hand‐held homogenizer on ice. Doxorubicin standard homogenates were prepared by adding different concentrations of doxorubicin to the tissue homogenates of each organ from the nontreated mice. The homogenized samples were then spiked with daunorubicin (20 ng) as an internal standard and incubated for 15 min while vortexed at room temperature. 3 m AgNO_3_ (50 µL) was added to the homogenate and vortexed for 5 min followed by addition of 3 m NaCl (50 µL) and 5 min of vortex. Then, 1.25 mL of a mixture of acetonitrile and methanol (2:1) was added, vortexed for 10 min, and sonicated for 10 min. The samples were centrifuged for 10 min at 17 500 × g and the supernatants were collected. The supernatants were concentrated to near 100 µL using a centrifugal evaporator. The concentrated samples were centrifuged for 10 min at 17 500 × g and the supernatants were removed. Acetonitrile (200 µL) was added to the residue and sonicated for 10 min. Undissolved residue was removed by centrifuge for 10 min at 17 500 × g and the samples were subjected to HPLC analysis. The samples were subjected to an analytical HPLC (Agilent 1300 series) equipped with an ODS‐A 5 µm analytical column (150 mm × 3 mm; YMC) at an injection volume of 100 µL. A gradient system (water and ACN with 0.1% TFA as an additive, ACN 10–90%/5–25 min) was applied at a flow rate of 1 mL min^−1^. The peaks were monitored under a fluorescence detector at 470/580 nm.

*Toxicity Assessments*: The normal 6‐week‐old male ICR mice (Orient Bio) received one of the followings once a day for seven days (*n* = 10): normal saline, doxorubicin (3 mg kg^−1^), and RGDEVD‐DOX (3 mg kg^−1^ doxorubicin molar equivalent). During the drug treatment, body weight was measured. At the day of study termination, sodium citrate‐stabilized whole blood and serum were collected, and the major organs were harvested. The blood tests were conducted by Seegene Medical Foundation (Seoul, South Korea). The organs were formalin‐fixed, paraffin‐embedded, and sectioned for hematoxylin & eosin staining.

*Statistical Analysis*: Data are presented as means ± s.d. Statistical methods were not used to estimate the necessary sample size. Every data obtained were included in the analysis. Experiments were performed nonblinded. The Kolmogorov–Smirnov (for *n* ≥ 5) and Shapiro–Wilk (for *n* ≥ 3) normality tests were used to determine the normality of the data. For normally distributed data, Student's *t*‐test (with Welch's correction when the variance between groups was different) or one‐way ANOVA with Holm–Sidak post hoc test was used to determine statistical differences between two groups and among multiple groups, respectively. The variance among groups was found similar by *F*‐test or Brown–Forsythe test. For the non‐normally distributed data, Kruskal–Wallis test was used to determine statistical differences among multiple groups. Log‐rank test was used to determine the statistical differences of overall survival results. All statistical analyses were two‐sided and *P* values below 0.05 were considered statistically significant. Statistical calculations were performed using GraphPad Prism 7 (GraphPad Software, San Diego, CA).

## Conflict of Interest

The authors declare no conflict of interest.

## Supporting information

Supplementary
