# Supplementary material for: Self‐Triggered Apoptosis Enzyme Prodrug Therapy (STAEPT): Enhancing Targeted Therapies via Recurrent Bystander Killing Effect by Exploiting Caspase‐Cleavable Linker
Source: Adv Sci (Weinh). 2018 Jun 5;5(7):1800368. doi: 10.1002/advs.201800368 (PMC6051143; doi:10.1002/advs.201800368)
Supplement: Supplementary file 1 — Supplementary [file ADVS-5-1800368-s001.pdf]

Copyright WILEY-VCH Verlag GmbH & Co. KGaA, 69469 Weinheim, Germany, 2018.

ADVANCED SCIENCE
Open Access

Supporting Information

for *Adv. Sci.,* DOI: 10.1002/advs.201800368

Self-Triggered Apoptosis Enzyme Prodrug Therapy (STAEPT): Enhancing Targeted Therapies via Recurrent Bystander Killing Effect by Exploiting Caspase-Cleavable Linker

Seung Woo Chung, Jeong Uk Choi, Young Seok Cho, Ha Rin Kim, Tae Hyung Won, Peter Dimitrion, Ok-Cheol Jeon, Seong Who Kim, In-San Kim, Sang Yoon Kim, and Youngro Byun**

Copyright WILEY-VCH Verlag GmbH & Co. KGaA, 69469 Weinheim, Germany, 2016.

Supporting Information

Self-Triggered Apoptosis Enzyme Prodrug Therapy (STAEPT): Enhancing Targeted Therapies via Recurrent Bystander Killing Effect by Exploiting Caspase-Cleavable Linker

Seung Woo Chung, Jeong Uk Choi, Young Seok Cho, Ha Rin Kim, Tae Hyung Won, Peter Dimitrion, Ok-Cheol Jeon, Seong Who Kim, In-San Kim, Sang Yoon Kim*, and Youngro Byun*

Table S1. Hematological and biochemical analysis of blood samples from ICR mice that received doxorubicin and RGDEVD-DOX at 3 mg kg^{-1} (dox molar eq) once a day for seven days (n = 5).

Parameters	Unit	Control	Doxorubicin	RGDEVD-DOX
Biochemistry				
ALT	U/L	18 ± 7	66 ± 46	16 ± 5
AST	U/L	73 ± 21	80 ± 23	53 ± 9
ALP	U/L	289 ± 51	133 ± 46	222 ± 25
BUN	mg/dL	20.0 ± 2.1	9.7 ± 2.2	17.2 ± 3.0
CRE	mg/dL	0.3 ± 0.0	0.3 ± 0.1	0.2 ± 0.1
TP	g/dL	4.6 ± 0.3	2.8 ± 0.7	4.0 ± 0.2
ALB	g/dL	2.0 ± 0.2	1.2 ± 0.3	1.7 ± 0.1
TBIL	mg/dL	0.7 ± 0.1	0.6 ± 0.4	0.6 ± 0.1
CK	U/L	482 ± 338	279 ± 106	176 ± 66
CK-MB	U/L	187 ± 65	324 ± 94	190 ± 82
LDH	U/L	1420 ± 336	2775 ± 1013	1099 ± 233
Hematology				
WBC	K/μL	3.29 ± 0.47	1.80 ± 0.38	2.97 ± 0.28
RBC	M/μL	7.98 ± 0.27	7.69 ± 0.61	7.57 ± 0.14
HGB	g/dL	13.0 ± 0.4	12.4 ± 1.0	12.3 ± 0.3
HCT	%	43.7 ± 1.4	38.8 ± 3.0	41.6 ± 0.9
MCV	fL	54.8 ± 1.1	50.5 ± 0.3	53.7 ± 1.1
MCH	pg	16.2 ± 0.4	16.1 ± 0.3	16.2 ± 0.3
MCHC	g/dL	29.6 ± 0.4	31.8 ± 0.4	30.4 ± 0.9
PLT	K/μL	1026 ± 74	1296 ± 214	1166 ± 96
LYMPH	%	79.1 ± 6.7	70.1 ± 4.0	72.1 ± 8.1
MONO	%	2.5 ± 0.7	1.3 ± 0.4	3.1 ± 1.5
EOS	%	1.6 ± 0.8	1.2 ± 0.3	1.2 ± 0.2
BASO	%	0.5 ± 0.3	0.4 ± 0.3	0.5 ± 0.2
RDW	%	14.0 ± 0.8	13.8 ± 0.2	14.3 ± 0.2
MPV	fL	6.1 ± 0.2	5.7 ± 0.1	6.2 ± 0.4

ALT, alanine transaminase; AST, aspartate aminotransferase; ALP, alkaline phosphatase; BUN, blood urea nitrogen; CRE, creatinine; TP, total protein; ALB, albumin; TBIL, total bilirubin; CK, creatine kinase; CK-MB, creatine kinase MB isoenzyme; LDH, lactate dehydrogenase; WBC, white blood cell; RBC, red blood cell; HGB, hemoglobin; HCT, hematocrit; MCV, mean corpuscular volume; MCH, mean corpuscular hemoglobin; MCHC, mean corpuscular hemoglobin concentration; PLT, platelet; LYMPH, lymphocyte; MONO, monocyte; EOS, eosinophil; BASO, basophil; RDW, red cell distribution width; MPV, mean platelet volume. Data are presented as mean ± s.d.

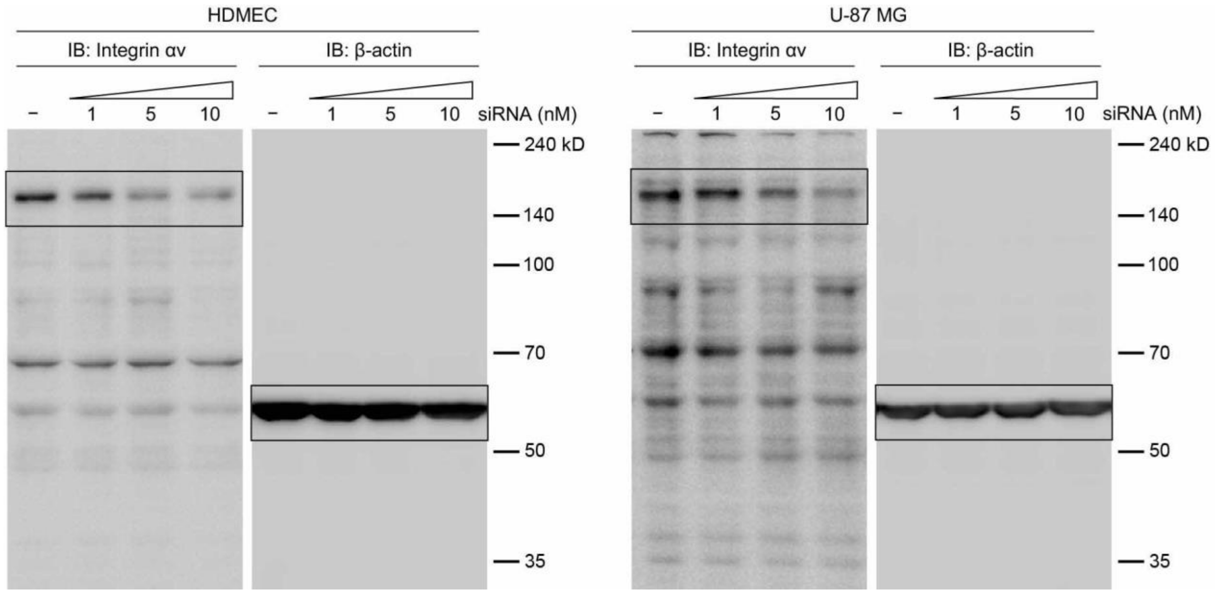

Figure S1. Full length western blots of integrin αv in HDMECs and U-87 MG cells transfected with different concentrations of ITGAV siRNA. The box indicates the location of the bands in interest. The cells transfected with 10 nM of ITGAV siRNA were used for further experiments.

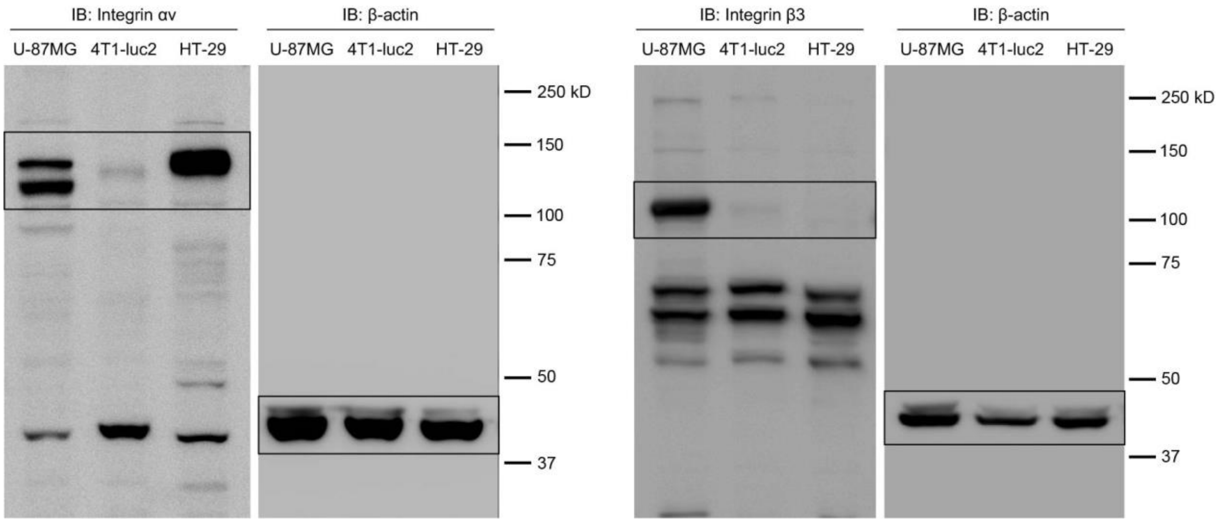

Figure S2. Full length western blots of integrin αv (left) and integrin β3 (right) in U-87 MG, 4T1-luc2, and HT-29. The box indicates the location of the bands in interest.

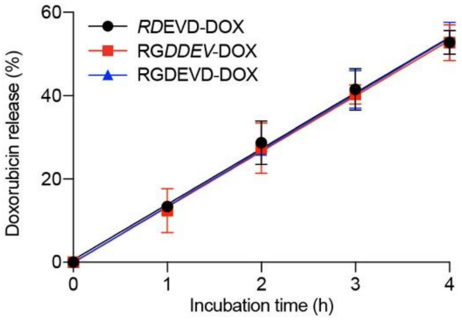

Figure S3. Doxorubicin release from RDEVD-DOX, RGDEVD-DOX, and RGDDEV-DOX when incubated in PBS (pH 7.4) containing carboxylesterase (n = 3). Data are mean ± s.d.

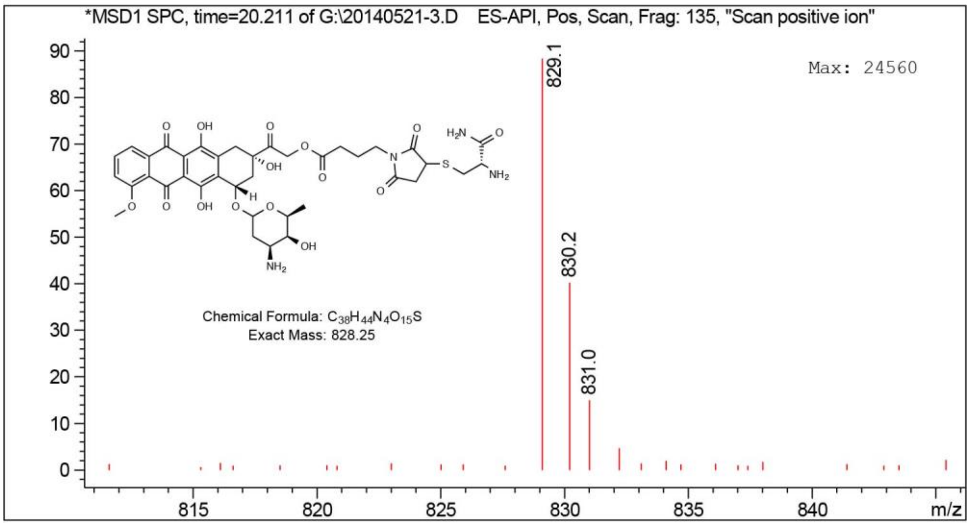

Figure S4. Mass spectrum of the active compound released from RGDEVD-DOX after incubation with recombinant human caspase-3. Chemical structure, chemical formula, and expected mass value are also presented.

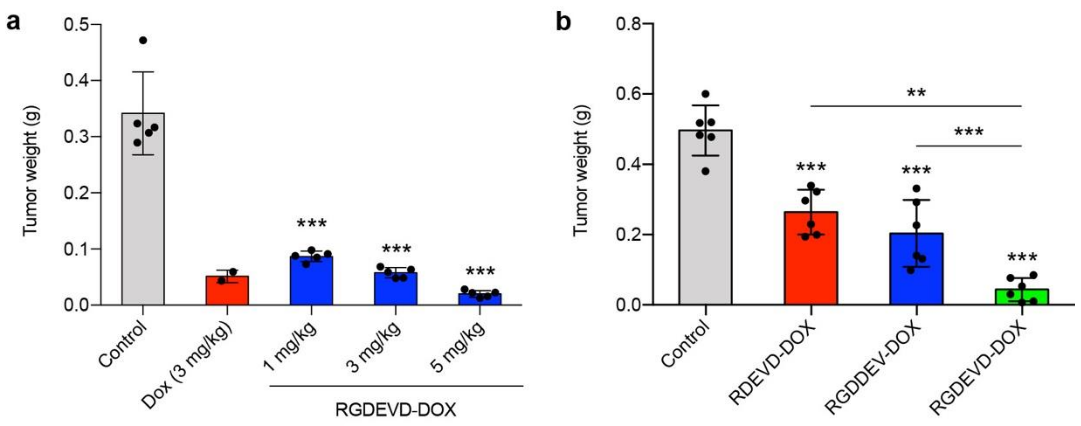

Figure S5. Anticancer activity of RGDEVD-DOX in U-87 MG xenografts. a) Tumor weight of mice that received normal saline as control, doxorubicin (3 mg kg^{-1}), or RGDEVD-DOX (1, 3, 5 mg kg^{-1}; n = 5). b) Tumor weight of mice that received normal saline as control, RGDEVD-DOX, RDEVD-DOX, or RGDDEV-DOX (n = 6) at 3 mg kg^{-1}. Drugs were administered once a day for seven days and the tumors were harvested seven days after the last treatment. Doses are presented as doxorubicin molar equivalent dose. Data are mean ± s.d. $^{**}P < 0.01$, $^{***}P < 0.001$.

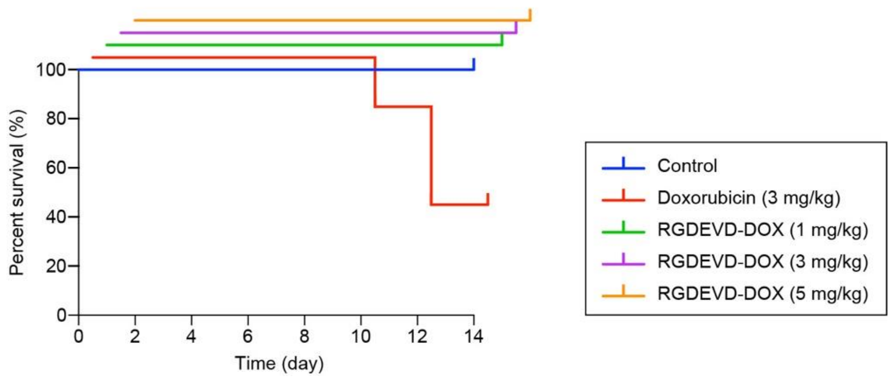

Figure S6. Kaplan-Meier survival curve of U-87 MG xenografted mice that received normal saline as control, doxorubicin (3 mg kg^{-1}), or RGDEVD-DOX (1, 3, 5 mg kg^{-1}) once a day for seven days (n = 5). Doses are dox molar equivalent dose.

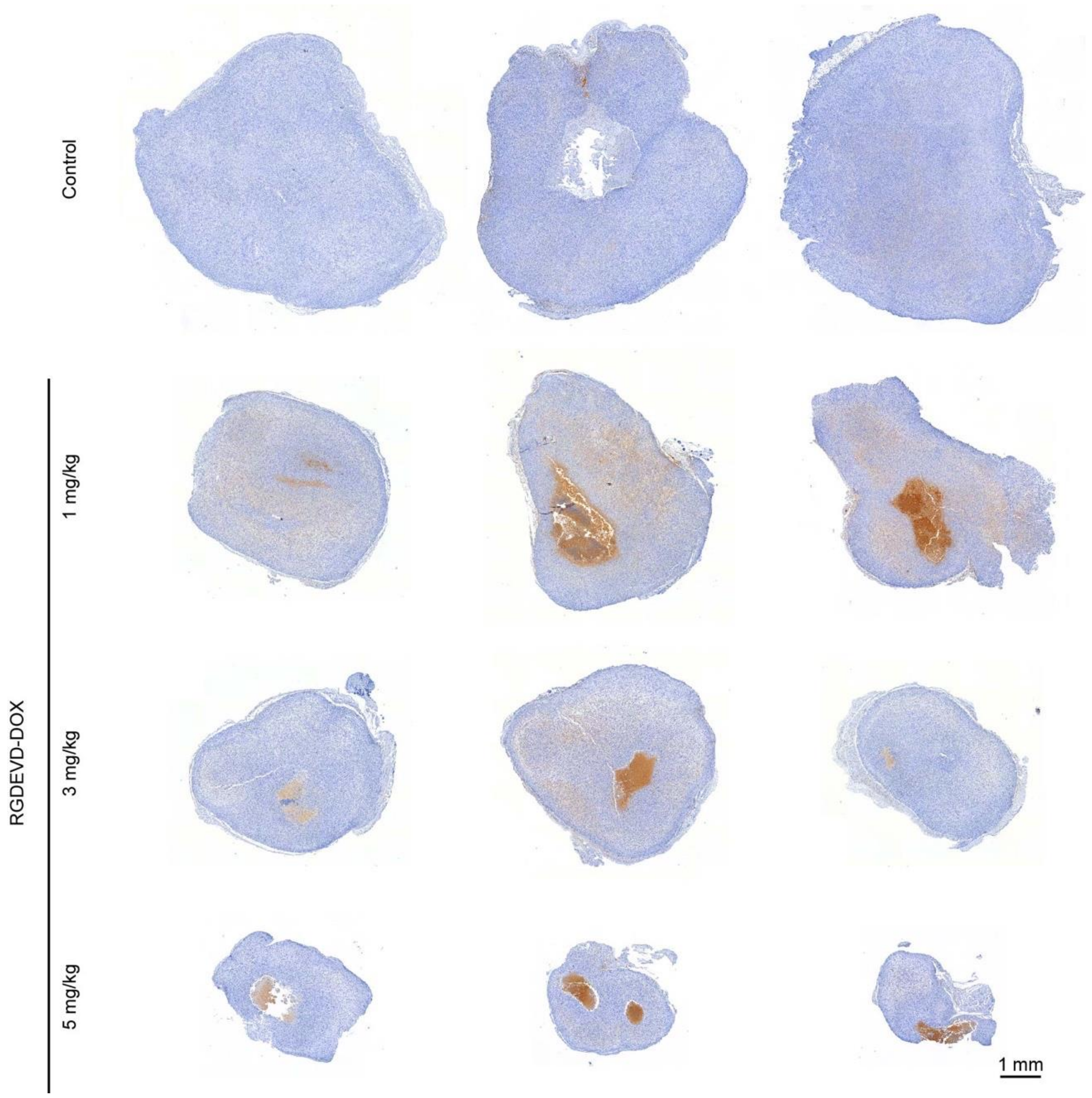

Figure S7. Full panel images of immunohistochemical staining of caspase-3 in the tumor sections from the U-87 MG xenografted mice that received RGDEVD-DOX in different doses for 7 days. Scale bar, 1 mm.

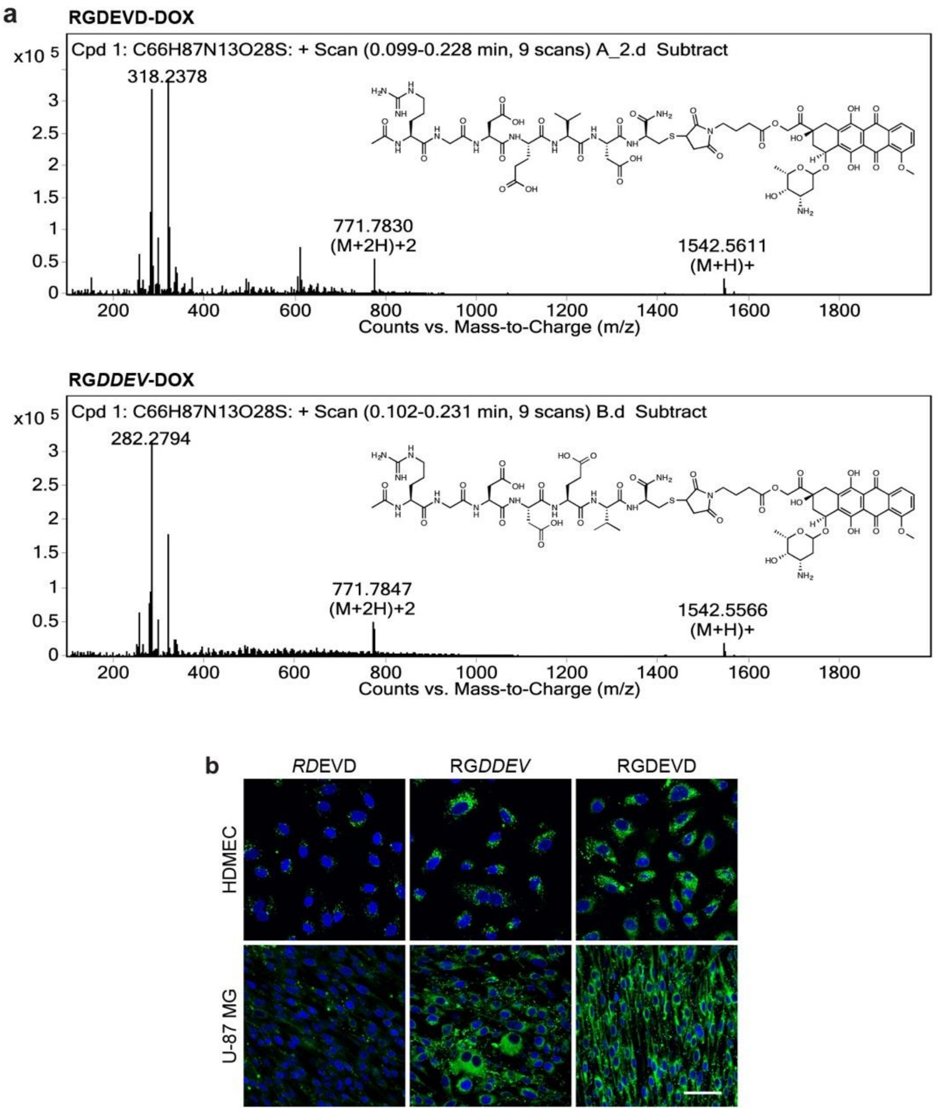

Figure S8. Characterization of the DEVD-deficient prodrug analog, RGDDEV-DOX. a) Chemical structure and high-resolution mass spectrum RGDEVD-DOX (upper) and RGDDEV-DOX (lower). b) The cellular uptake of fluorescent-labeled RDEVD, RGDEVD, and RGDDEV peptides in integrin αvβ3-overexpressing HDMEC and U-87 MG cells. Scale bar, 50 μm.

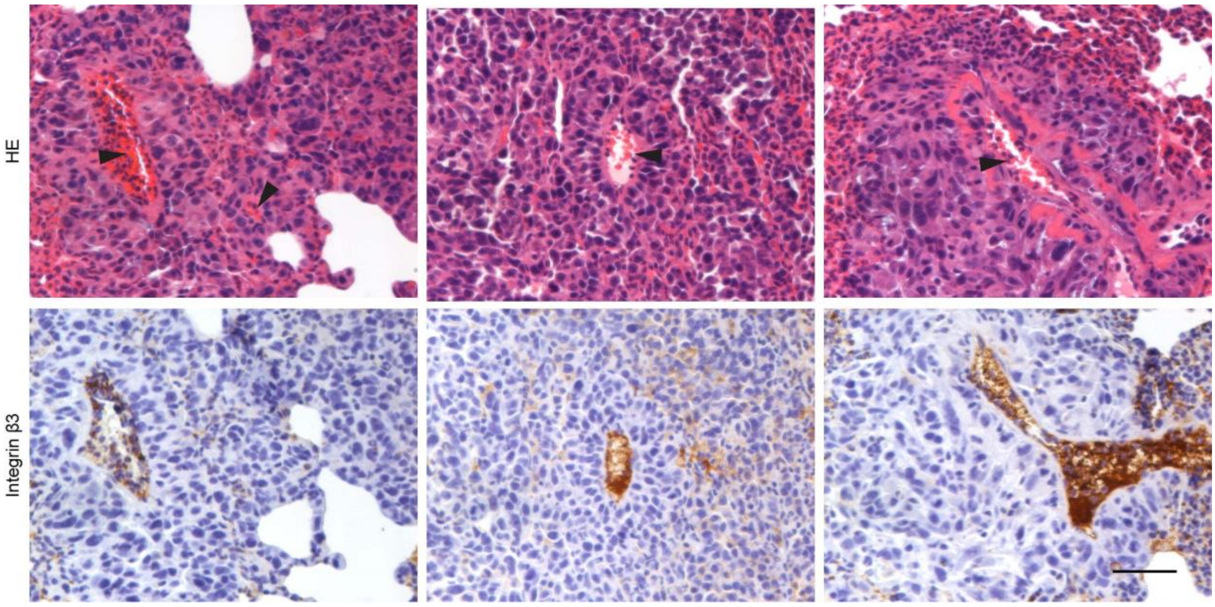

Figure S9. HE and immunohistochemical staining of integrin β3 in 4T1-luc2 lung metastases. Scale bar, 50 µm. Arrowheads indicate red blood cells in the blood vessels.

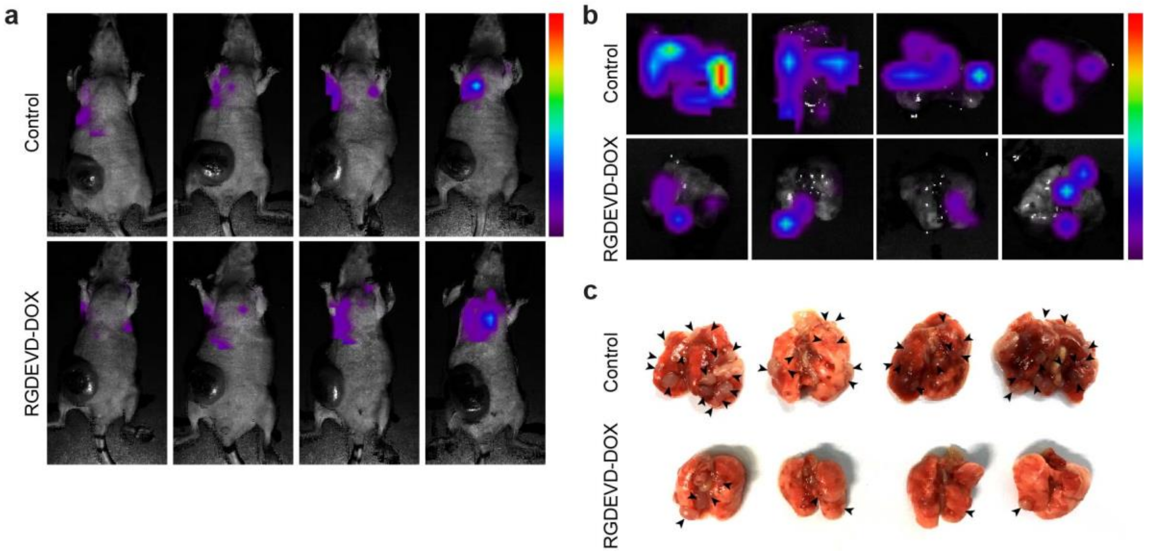

Figure S10. a) Full panel bioluminescence images of 4T1-luc2 lung metastases before drug treatment. Full panel images of the b) bioluminescence and c) surface tumor nodules of 4T1-luc2 metastasized lung of mice treated with normal saline as control or the prodrug. Arrowheads indicate tumor nodules on the lung. Drug administration, 3 mg kg^{-1} dox molar equivalent once a day for seven days via the intravenous route.

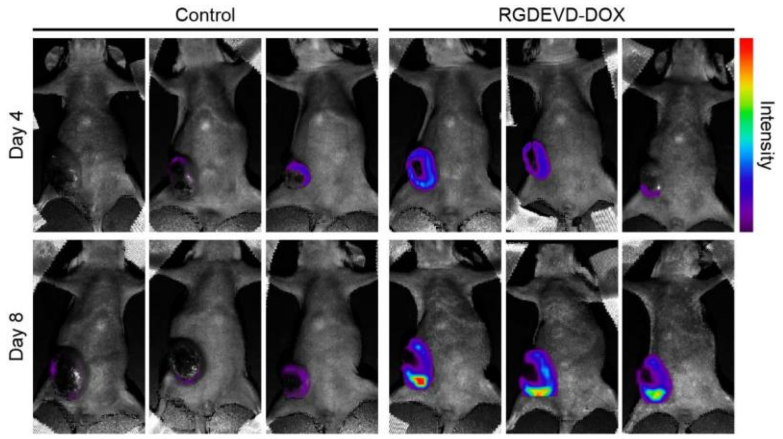

Figure S11. Full panel bioluminescence images of caspase-3 expression in the 4T1-luc2 primary tumor at day 4 and 8 during the administration of normal saline as a control or the prodrug (n = 3). Drug administration, 3 mg kg^{-1} dox molar equivalent once a day for seven days via the intravenous route.
